# Role of Calpains in Uremia-Related Functional and Structural Muscle Changes: Protective Effect of Calpastatin Overexpression

**DOI:** 10.3390/cells14231846

**Published:** 2025-11-23

**Authors:** Elena Gutiérrez-Calabrés, Sofía Campillo, Elena Alcalde-Estévez, Paula Cuevas-Delgado, Coral Barbas, Sergio García-Villoria, Alba Silvestre-Vargas, Mercedes Griera, Sergio de Frutos, María P. Ruiz-Torres, Diego Rodríguez-Puyol, Laura Calleros

**Affiliations:** 1 Physiology Unit, Department of Systems Biology, Universidad de Alcalá, 28871 Alcalá de Henares, Madrid, Spain; sofia.campillo@uah.es (S.C.); elena.alcaldee@uah.es (E.A.-E.); sergio.garciavillori@uah.es (S.G.-V.); alba.silvestre@uah.es (A.S.-V.); mercedes.griera@uah.es (M.G.); sergio.frutos@uah.es (S.d.F.); mpiedad.ruiz@uah.es (M.P.R.-T.); laura.calleros@uah.es (L.C.); 2 Fundación Renal Española, 28871 Madrid, Spain; diego.rodriguez@uah.es; 3 Instituto Ramón y Cajal de Investigación Sanitaria (IRYCIS), 28871 Madrid, Spain; 4 INNOREN-CM, 28871 Madrid, Spain; 5 RICORS2040 Kidney Disease, Instituto de Salud Carlos III, 28871 Madrid, Spain; 6 Centre for Metabolomics and Bioanalysis (CEMBIO), Chemistry and Biochemistry Department, Pharmacy Faculty, Universidad San Pablo-CEU, 28871 Boadilla del Monte, Madrid, Spain; pau.cuevas.ce@ceindo.ceu.es (P.C.-D.); cbarbas@ceu.es (C.B.); 7 Department of Medicine and Medical Specialties, Universidad de Alcalá, 28871 Alcalá de Henares, Madrid, Spain; 8 Biomedical Research Foundation and Nephrology Unit, Hospital Universitario Príncipe de Asturias, 28871 Alcalá de Henares, Madrid, Spain

**Keywords:** chronic kidney disease, sarcopenia, calpain, calpastatin, muscle proteolysis

## Abstract

**Highlights:**

**What are the main findings?**

- Adenine-induced CKD leads to a decline in muscle strength and deterioration of muscle quality, even in the absence of significant muscle mass loss.
- Uremic conditions upregulate CAPN2 expression and activity in skeletal muscle, suggesting a pathogenic role in muscle deterioration.
- Inhibition of calpain activity through CAST overexpression preserves muscle function and attenuates fibrosis, inflammation and adipogenic processes.
- The protective effects of CAPN inhibition occur independently of improvements in renal function.

**What is the implication of the main finding?**

- CAPN2 emerges as a potential therapeutic target to prevent sarcopenia in CKD.

**Abstract:**

Sarcopenia, the progressive loss of muscle mass and strength, is a common complication in patients with chronic kidney disease (CKD). This condition arises from a combination of factors including reduced physical activity, insufficient protein intake, hyperphosphatemia, chronic inflammation, and uremia itself; however, the underlying molecular mechanisms remain poorly understood. Proteolysis in skeletal muscle is primarily controlled by the ubiquitin–proteasome system, autophagy–lysosome system, and calpains (CAPNs) cysteine proteases, which degrade structural proteins and mediate cell signaling. This study aims to investigate the role of CAPNs in CKD-associated muscle deterioration. CKD was induced in mice through an adenine-rich diet for 2, 4 and 6 weeks. The involvement of CAPNs in CKD-related sarcopenia was assessed using mice that overexpressed the CAPNs endogenous inhibitor, calpastatin (CAST). Gastrocnemius muscle strength, structural integrity, and function were evaluated. Mice with CKD showed elevated CAPNs, particularly CAPN2, expression and activity in the gastrocnemius, in parallel with significant muscle deterioration, including strength loss, structural damage, and impaired muscle performance. Overexpression of CAST prevented muscle strength loss, improved muscle function and structure without affecting renal function, and reversed fibrosis, inflammation and adipogenesis expression markers. Targeting CAPN2 could be a promising therapeutic strategy to mitigate muscle damage and improve physical performance in CKD patients.

## 1. Introduction

Sarcopenia, defined as a progressive and generalized loss of muscle mass and strength [1], is a common complication in patients with chronic kidney disease (CKD) [2]. This condition is associated with a gradual decline in physical performance, negatively impacting quality of life and clinical outcomes in this population. The loss of muscle mass generally occurs due to the combination of two factors: muscle atrophy and the death of muscle cells. At the cellular level, atrophy is caused by a deregulation of protein turnover in which degradation is greater than synthesis. Proteolysis in skeletal muscle is primarily regulated by the ubiquitin–proteasome system and involves the autophagy–lysosome system and proteases such as calpains (CAPNs) [3]. It has been observed that when muscle atrophy occurs, the expression of two ubiquitin ligases, atrogin-1 and MuRF-1, increases, which triggers the degradation of muscle proteins [4]. Overall, sarcopenia is a consequence of disuse atrophy.

The etiological factors involved in sarcopenia among patients with CKD are diverse and may be associated with several conditions, including the kidney disease itself, the dialysis procedure, and the low-grade chronic inflammation commonly present in these patients. It has been observed that the uremic environment increases oxidative stress in stem cells from CKD patients, leading to reduced viability, decreased proliferative capacity, and disruptions in the respiratory chain [5]. Furthermore, increasing concentrations of the uremic toxin indoxyl sulfate (IS) may cause a higher degree of apoptosis in skeletal muscle myoblasts [6] and uremic toxins reduce the functional capacity of myoblasts, decreasing cell differentiation and myotube formation [7]. In patients undergoing end-stage kidney disease, elevated serum levels of IS are associated with reduced muscle strength [8]. Studies performed in gastrocnemii from uremic rats showed higher collagen expression and lower myosin heavy chain expression than those from healthy rats [5]. On the other hand, systemic increases in phosphate levels (hyperphosphatemia) which are common in CKD [9], may decrease calcium release from the sarcoplasmic reticulum, affecting muscle contraction. Studies performed in the C_2_C_12_ cell model showed that phosphate treatment inhibits myoblast differentiation, induces senescence and reduces proliferative capacity [10,11]. In addition, in myotubes phosphate induces oxidative stress and alters mitochondrial function [12].

CAPN is a calcium-dependent intracellular cysteine protease first described in 1964 by Gordon Guroff [13]. There are 15 CAPN genes in the human genome, two genes encoding regulatory subunits (CAPNS1 and CAPNS2), and one for calpastatin (CAST) [14]. CAPNs 1 and 2 were the first two members of this family to be described. They are primarily localized in the cytosol, are almost ubiquitously expressed, and exhibit Ca^2+^-dependent proteolytic activity at neutral pH. They require micromolar (3 to 50 μM) and millimolar (0.2 to 1 mM) concentrations of Ca^2+^ in vitro to become active; that is why they were originally named μ- and m-calpain, respectively [15].

CAPNs play a pivotal role in the onset of sarcopenia. According to some researchers, apoptosis associated with sarcopenia might involve CAPN-dependent proteolysis rather than caspase-dependent mechanisms [16]. To reinforce this idea, CAPN has been localized in mitochondria and, in addition, several CAPN substrates have been identified among the components of the respiratory chain [17]. Furthermore, the proapoptotic factors AIF and EndoG, which can be released from mitochondria by CAPN activity, are upregulated in sarcopenic muscle [18]. On the other hand, an abnormal increase in CAPN activity could explain the degradation and necrosis of myofibrils, as well as the impairment in the proliferation and migration of SCs observed in aged-related sarcopenic muscle [19].

Taken together, these results would suggest that the proteolytic function of CAPNs could contribute to the appearance of extrarenal complications associated with CKD, such as sarcopenia. Therefore, the different isoforms of CAPNs could be useful as therapeutic targets in sarcopenic patients. Despite studies on the involvement of CAPNs in sarcopenia, the role of these proteases in the genesis of muscular complications associated with CKD has not yet been fully described.

In this study, we used a model of CAST overexpression [20] to investigate the role of calpains in CKD-associated skeletal muscle deterioration. To this end, mice were subjected to an adenine-rich diet to induce chronic kidney damage and reproduce the uremic environment characteristic of CKD [21].

## 2. Materials and Methods

### 2.1. Animal Model and Study Design

All animal procedures were previously approved by the Institutional Animal Care and Use Committee of the University of Alcalá and conformed to Directive 2010/63/EU of the European Parliament and the ARRIVE reporting guidelines. No specific inclusion criteria were applied. Animals were excluded if genotyping results did not match the assigned experimental group or if the animal’s health status was compromised, as assessed in accordance with the severity criteria outlined in the approved project license. The ethical approval for the study was obtained on 29 July 2022, under the approval code PROEX 160.7/22. These exclusion criteria were defined a priori. All animals were housed in the same pathogen-free and temperature-controlled room (22 °C ± 2 °C). Food and water were available ad libitum. Cage positions on the rack were fixed throughout the experiment. To reduce the impact of potential confounding variables such as cage location or time of day, animals were handled and sacrificed in a random order each day. Testing and euthanasia were carried out between 9:00 a.m. and 13:00 p.m., with the daily order of animals randomized to avoid systematic bias. CKD was induced in 3- or 4-month-old male and female C57BL/6 mice by feeding a diet containing 0.2% adenine (ssniff Spezialdiäten GmbH, Soest, Germany) as previously described [21] for 2, 4 and 6 weeks. Humane endpoints and monitoring criteria were defined in the approved project license (PROEX 160.7/22). Animals were monitored accordingly by trained personnel throughout the study. No adverse events were observed during the study.

To study the role of CAPNs in CKD-related sarcopenia, we used a CAST overexpression model (Infrafrontiers EMMA repository [20]). To detect the rabbit CAST transgene (518 bp), tail DNA was genotyped by PCR with primers GTTGGCTTAGGCTGCTTTTCGT and CCAGACTCCGTGACACCCCTT [20]. PCR DNA products were then analyzed using 1% agarose gel electrophoresis. For the induction of CKD, WT and CAST mice were bred as littermate controls and fed an adenine-containing diet for 4 weeks.

After treatment had finished, mice were anesthetized, and peripheral blood was extracted intracardially and collected in tubes with 0.1% EDTA as an anticoagulant. Plasma was separated by centrifugation at 3000 rpm for 15 min and stored at −80 °C until assayed. Muscle mass was measured by the weight of gastrocnemius normalized to body mass. Gastrocnemius muscles were collected in 4% paraformaldehyde for Sirius red staining. Samples were also stored in RNAlater solution (Life Technologies, Carlsbad, CA, USA) at −80 °C for the Western blot, RT-qPCR and CAPN activity assays. The individual mouse was considered the experimental unit within the studies. Although no formal a priori sample size calculation was conducted, the number of animals used in each experimental group (typically between 6 and 12) was determined based on prior experience with similar models, adherence to relevant ethical and legal regulations, including Directive 2010/63/EU on the protection of animals used for scientific purposes, and in accordance with the 3Rs principle. This range was also sufficient to allow for sex-disaggregated analyses when needed. Littermate mice were randomly divided into control and treatment groups by generating random numbers using the standard = RAND() function in Microsoft Excel. Group allocation and experimental procedures were carried out by two researchers who were aware of the group assignments. To reduce bias, outcome assessment and data analysis were performed by an independent researcher blinded to group allocation.

### 2.2. Plasma Determinations

Urea nitrogen (EIABUN; Invitrogen, Thermo Fisher Scientific; Waltham, MA, USA) and plasma creatinine (Cayman Chemical; Ann Arbor, MI, USA) were measured using colorimetric assay kits, according to the manufacturer’s instructions. The spectrophotometric measurements were performed in a Victor X4 Multilabel Plate Reader (PerkinElmer, Waltham, MA, USA) at a wavelength of 450 nm (plasma urea nitrogen) and 490 nm (plasma creatinine).

Plasma phosphorus concentration was quantified by a colorimetric assay using the commercial kit QuantiChrom Phosphate Assay (DIPI-500) (BioAssay Systems; Hayward, CA, USA) following the manufacturer’s instructions. The absorbance was analyzed at a wavelength of 620 nm using an EL800 plate reader (BioTek, Agilent Technologies, Waldbronn, Germany).

The determination of free and total IS and p-cresyl sulfate (pCS) concentrations was performed at the Metabolomics and Bioanalysis Center (CEMBIO) of Universidad San Pablo-CEU using ultra-high-performance liquid chromatography–tandem mass spectrometry (UHPLC-MS/MS) with slight modifications to a previously described method [22]. Plasma samples were treated using protein precipitation with acetonitrile containing stable isotope-labeled pCS-D7 as an internal standard. For free IS and pCS, plasma ultrafiltration was performed, and the resulting filtrate followed the same processing method described above. For both analyses, 5 µL of sample was injected, and chromatographic separation was achieved with an Acquity UPLC^®^ BEH C18 column (1.7 µm, 2.1 mm × 100 mm) and a Vanguard Acquity UPLC^®^ BEH C18 pre-column (2.1 × 5 mm), maintained at 30 °C in the UHPLC system oven (1290 Infinity, Agilent Technologies, Waldbronn, Germany). The mobile phase consisted of 0.1% formic acid (*v*/*v*) in water for phase A and 0.1% formic acid (*v*/*v*) in acetonitrile for phase B. Analyte concentrations were calculated from the calibration curve of ion ratios between the analytes and the internal standard.

### 2.3. Muscle Strength and Physical Performance

Gait parameters were assessed during spontaneous mouse locomotion along a 60 cm long, 3 cm wide catwalk covered with absorbent paper. Non-toxic ink was used to stain the mice’s footprints. Following a familiarization period, each mouse completed four unassisted walks, with 5 min intervals between trials. Gait speed was calculated using time and distance, while stride length and hind paw base width were determined by analyzing the spatial pattern of the ink footprints.

Orientation and motor coordination were tested on a 60 cm long, 28 mm diameter wooden rod fixed 60 cm above a padded surface. A mark 10 cm from the supported end served as the finish line. Mice were placed at the “open” end of the rod, facing away from the bench. After familiarization, each mouse underwent three trials with 5 min intervals between them. Two parameters were recorded: orientation time (time taken to turn 180° to face the supported end) and transition time (time taken to reach the finish line). If a mouse fell twice during orientation or transition, a default score of 25 s was assigned.

Forelimb grip strength was measured using a Grip Strength Meter (UGO BASILE, purchased from PSYMTEC, Madrid, Spain). For the test, the mouse was placed in front of a grasping bar connected to a force transducer and amplifier. The animal was allowed to grip the bar, then gently pulled backwards by its tail along a horizontal plane. Each mouse performed three trials of five consecutive attempts, with 1 min intervals between trials. Grip strength was calculated by dividing the mean peak force of all attempts by the mouse’s body weight.

### 2.4. Nuclear Magnetic Resonance Imaging

Skeletal muscle quality was evaluated using nuclear magnetic resonance imaging (NMRI). The experiments were conducted on a Bruker Pharmascan 7.0-T horizontal-bore system (Bruker Medical GmbH, Ettlingen, Germany) equipped with a 1H selective 23 mm birdcage resonator and a Bruker gradient insert (90 mm diameter, maximum intensity of 30 G/cm) at the Institute of Biomedical Research Alberto Sols CSIC-UAM (Madrid, Spain). Data was acquired using a Hewlett-Packard console running Paravision software 6.0.1 (Bruker Medical GmbH) on a Linux platform. The procedure was performed under general anesthesia with 2% isoflurane in O_2_. Mice were kept at 37 °C using a heated probe, and their respiratory rate was monitored with a Biotrig physiological monitor (Bruker Medical GmbH, Ettlingen, Germany).

### 2.5. Western Blot Analysis

Tissues were homogenized in lysis buffer (10 mM Tris–HCl, pH 7.6; 1% Triton X-100; 1 mM EDTA; 0.1% sodium deoxycholate) supplemented with protease and phosphatase inhibitors (Complete and PhosSTOP, Roche, Basel, Switzerland). Protein concentrations were determined by DC-Protein Assay (Bio-Rad, Hercules, CA, USA). Equal amounts of protein were separated on SDS–polyacrylamide gels and transferred to 0.2 μm-PVDF membranes (Bio-Rad, Hercules, CA, USA). Membranes were blocked and incubated with primary and secondary antibodies (Sigma-Aldrich or Dako, Glostrup, Denmark) afterwards. Primary antibodies used were against CAPN1, CAPN2, atrogin-1 (Abcam, Cambridge, UK) and GAPDH (Sigma-Aldrich, Glostrup, Denmark). Immunoblots were detected by chemiluminescence (Pierce ECL Western blotting Substrate, Thermo Fisher Scientific; Waltham, MA, USA) and imaged with ImageQuant LAS 500 System (General Electric Healthcare, Little Chalfont, UK). Densitometries were measured using ImageJ software 1.53 (National Institutes of Health, Bethesda, MA, USA).

### 2.6. Reverse Transcription–Quantitative Polymerase Chain Reaction (RT-qPCR)

After the corresponding experiments, the total RNA of tissue samples was extracted with TRIzol. Equal amounts of RNA were transcribed to cDNA with a High-Capacity cDNA Reverse Transcription Kit (Life Technologies, Carlsbad, CA, USA) and RT-qPCR analysis was performed in a 7500 qPCR thermocycler. TaqMan gene expression assays were used to quantify CAPN1 (Mm00482964_m1), CAPN2 (Mm00486669_m1), Fibronectin (Mm01256744_m1), TNFα (Mm00443258_m1), FABP4 (Mm00445878_m1) and GAPDH (Mm99999915_g1). Amplification values were normalized to endogenous GAPDH, and relative quantification was determined with 2^−ΔΔCT^ method.

### 2.7. Calpain Activity Assay

After the treatments, tissues were homogenized in lysis buffer (1 M Tris, 3 M NaCl and 0.1 M EDTA, pH 7.3) and protein concentrations were determined as previously described. Calpain activity was measured using the fluorogenic substrate Suc-LY-AMC (50 μM) (Calbiochem, Darmstadt, Germany) for 60 min at 37 °C. 50 µg (tissue) or 20 µg (cell) of protein were incubated with or without the pharmacologic calpain inhibitor Calpeptin (50 μM) (Calbiochem, Darmstadt, Germany) and in the presence of CaCl_2_ (10 mM). After incubation, intracellular calpain activity was measured spectrofluorometrically with a Victor X4 Multilabel Plate Reader with excitation at 380 nm and emission at 460 nm. Calpain activity was determined as the difference between fluorescence measured without and with Calpeptin.

### 2.8. Sirius Red Staining

Gastrocnemius tissue was fixed in 4% PFA, dehydrated and embedded in paraffin. Interstitial fibrosis was assessed by Sirius red staining of the collagen gastrocnemius content using the Picrosirius Red connective tissue staining kit (24901, Quimigen, Madrid, Spain). Paraffin muscle sections (4 μm) were deparaffinized, hydrated, and stained with phosphomolybdic acid solution, followed by Picrosirius Red and hydrochloric acid, according to the kit instructions. Eight random fields from each muscle section were photographed and the intensity of staining was analyzed with Image Pro-Plus software 5.1 (NIH) based on images from the microscope.

### 2.9. Statistical Analysis

All the data were analyzed using the GraphPad Prism software 7.0 (La Jolla, CA, USA). The results are presented as the mean ± standard error of the mean (SEM). Non-parametric statistics were used for comparisons, applying the Kruskal–Wallis test with Mann–Whitney post-test (non-paired data) or the Friedman test with Wilcoxon post-test (paired data). In both cases, Bonferroni correction was used. A *p*-value < 0.05 was considered statistically significant. Outlier detection was carried out using the Grubbs’ test and the determined values not included in the analysis. The sample size used in each group (6–12 animals) was based on our previous experience with the adenine-induced CKD model, where similar variability and effect sizes were observed [21]. This sample size has been shown to provide sufficient statistical power to detect biologically relevant differences [21], and to be in accordance with animal ethics and 3Rs guidelines.

## 3. Results

### 3.1. Functional and Structural Muscle Changes in Mice with Renal Damage Induced by Adenine Administration

**Figure 1:**
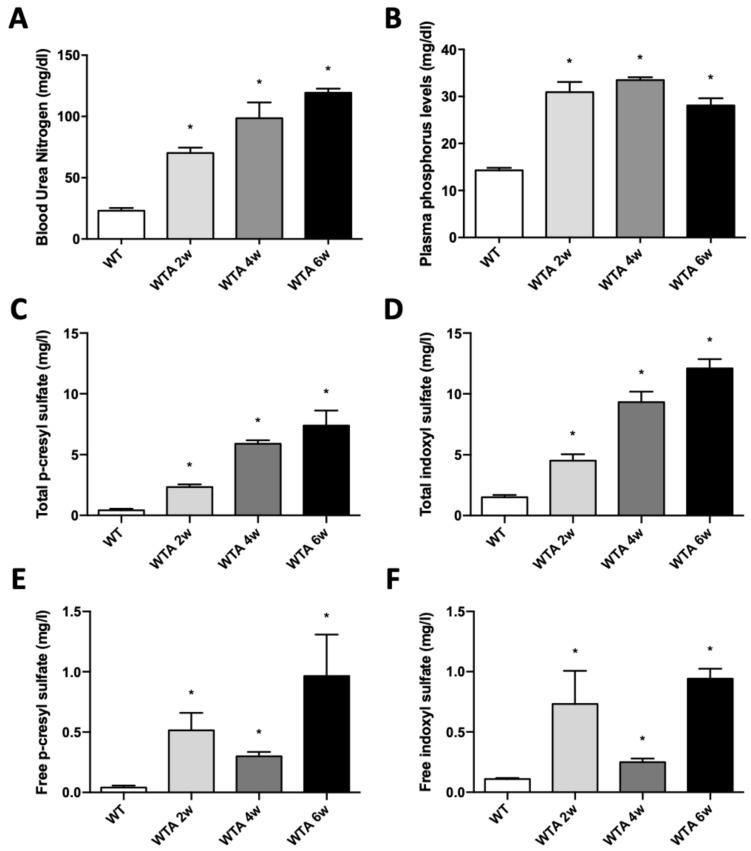
Adenine-rich diet induces kidney disfunction. Mice were fed for 2, 4 and 6 weeks with a standard (WT) or a 0.2% adenine-supplemented diet (WTA). (**A**–**C**): Bar graphs show the levels of BUN (blood urea nitrogen) (mg/dL) (**A**) and plasma phosphorus (mg/dL) (**B**), analyzed by colorimetric assays. (**C**–**F**): Bar graphs show total p-cresyl sulfate (pCS) (**C**) and indoxyl sulfate (IS) (**D**); and free pCS (**E**) and IS (**F**) plasma levels (mg/L), analyzed by UHPLC-MS /MS. All values are presented as the mean ± SEM. * *p* < 0.05 vs. WT. n: 6 animals/group.

As previously reported, mice receiving an adenine-containing diet (WTA) for 2, 4 and 6 weeks exhibited significant increased plasma urea (Figure 1A) and phosphorus (Figure 1B) concentrations; and increased pCS and IS plasma levels, both total and free (Figure 1C–F), compared with control animals (WT).

**Figure 2:**
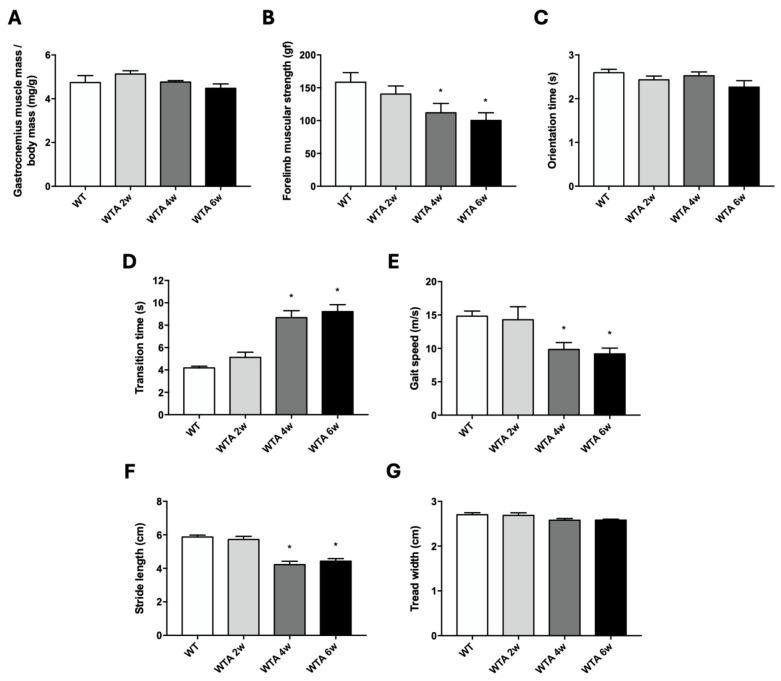
Adenine-rich diet decreases muscle strength and physical performance in mice. Mice were fed for 2, 4 and 6 weeks with a standard (WT) or a 0.2% adenine-supplemented diet (WTA). (**A**): Muscle mass was estimated analyzing the dry weight of gastrocnemius muscle and corrected by body weight (mg/g). (**B**): The bar graph shows the forelimb muscular strength (gf) measured by a grip strength test. (**C**–**E**): Bar graphs show orientation time (s) (**C**), transition time (s) (**D**) and gait speed (cm/s) (**E**), analyzed by a static bar test. (**F**,**G**): Bar graphs show stride length (cm) (**F**) and tread width (cm) (**G**) analyzed using a gait and stride analysis test. All values are presented as the mean ± SEM. * *p* < 0.05 vs. WT. n: 6–8 animals/group.

In terms of muscle mass and strength, even though no loss of gastrocnemius mass was detected (Figure 2A), a loss of forelimb muscular strength (Figure 2B) was observed in adenine-fed mice after 4 weeks of treatment compared to controls. When evaluating physical performance, a significant increase in transition time (Figure 2D) and a decrease in both gait speed (Figure 2E) and stride length (Figure 2F) were detected after 4 weeks of the adenine diet. However, no significant changes were observed in orientation time (Figure 2C) or tread width (Figure 2G) between the different experimental groups.

### 3.2. Role of Calpains in the Development of the Functional and Structural Muscle Changes Induced by Adenine Administration in Mice

**Figure 3:**
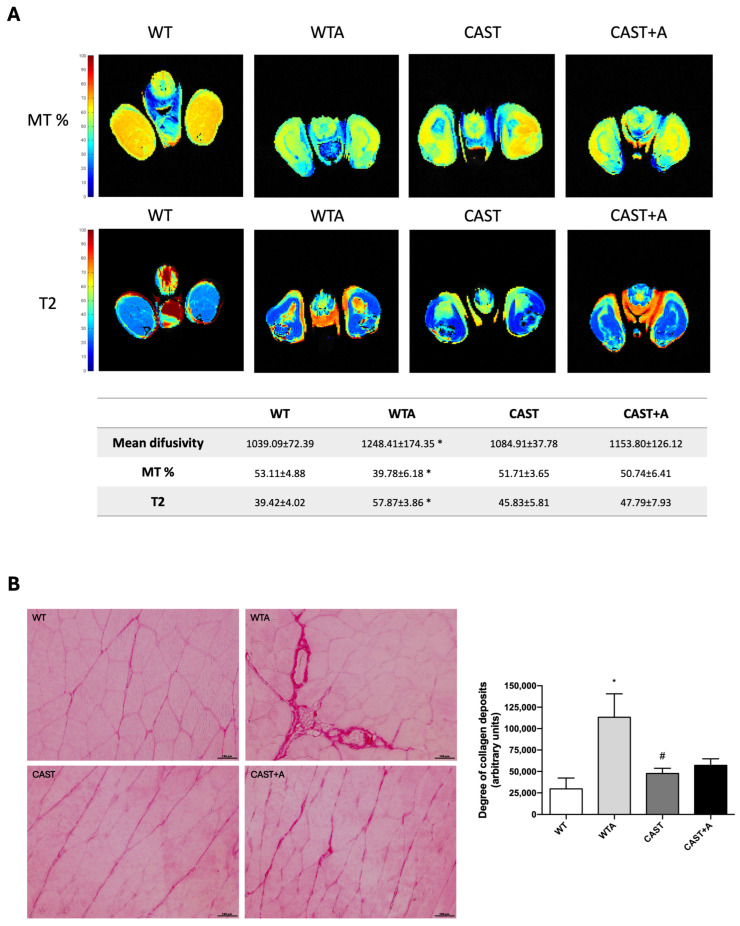
Calpastatin overexpression improves muscle structure damage and decreases gastrocnemius fibrosis in adenine-fed mice. Wild-type (WT) and calpastatin-overexpressing mice (CAST) were fed a standard or adenine-rich (WTA and CAST+A) diet for 4 weeks. (**A**): The mean diffusivity, percentage of magnetization transfer (MT%), and T2 relaxation time (T2) of the hind limbs were evaluated by nuclear magnetic resonance. An image of a representative experiment is shown. (**B**): Gastrocnemius collagen content was measured by Sirius red staining. An image of a representative experiment is shown. Scale bar: 100 µM. The bar graph represents the degree of collagen deposits. All values are presented as the mean ± SEM. * *p* < 0.05 vs. WT; # *p* < 0.05 vs. WTA. n: 6 animals/group.

Since significant changes in muscle function and structure were observed after 4 weeks of adenine administration, the following experiments were performed at that time. The structural muscle changes observed in mice after 4 weeks of treatment, summarized in the panels A and B of Figure 3, show that the adenine diet induced an increase in muscle hind limbs’ mean diffusivity, T2 relaxation time and collagen deposition (interstitial fibrosis); as well as a decreased percentage of magnetization transfer.

**Figure 4:**
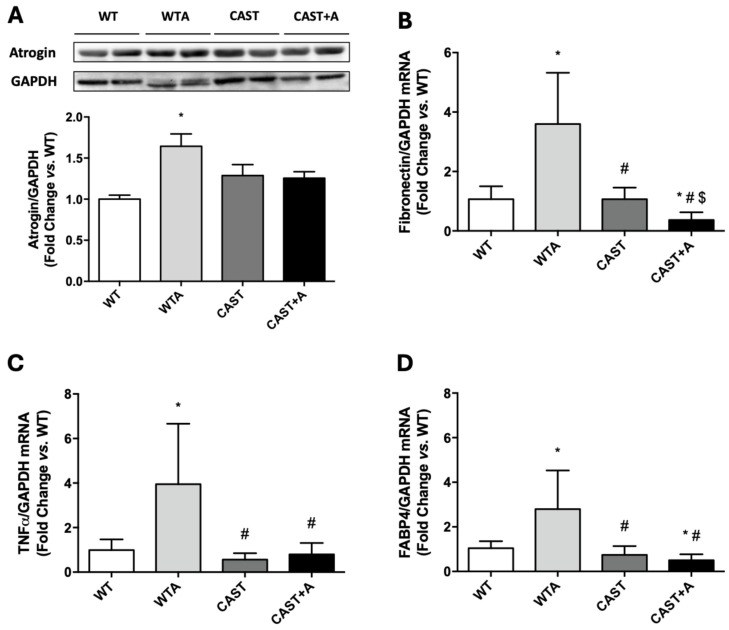
Calpastatin overexpression prevents muscle damage, fibrosis, inflammation and adiposis in the gastrocnemius of adenine-fed mice. Wild-type (WT) and calpastatin-overexpressing mice (CAST) were fed a standard or adenine-rich (WTA and CAST+A) diet for 4 weeks. (**A**): Atrogin protein content was evaluated by Western blot. An image of a representative blot is shown. The bars represent the normalized densitometric values of the blots against the endogenous control values versus WT. (**B**–**D**): Bar graphs show fibronectin (**B**), TNFα (**C**) and FABP4 (**D**) mRNA expression, analyzed by RT-qPCR. Relative changes in mRNA content versus WT are plotted after normalization to total GAPDH content as an endogenous control. All values are presented as the mean ± SEM. * *p* < 0.05 vs. WT; # *p* < 0.05 vs. WTA; $ *p* < 0.05 vs. CAST. n: 7–9 animals/group.

On the other hand, the adenine diet induced muscle damage analyzed by gastrocnemius atrogin content (Figure 4A), and increased mRNA levels of fibrosis (fibronectin), inflammation (TNFα) and adiposis (FABP4) after 4 weeks of treatment (Figure 4B–D), compared to WT.

**Figure 5:**
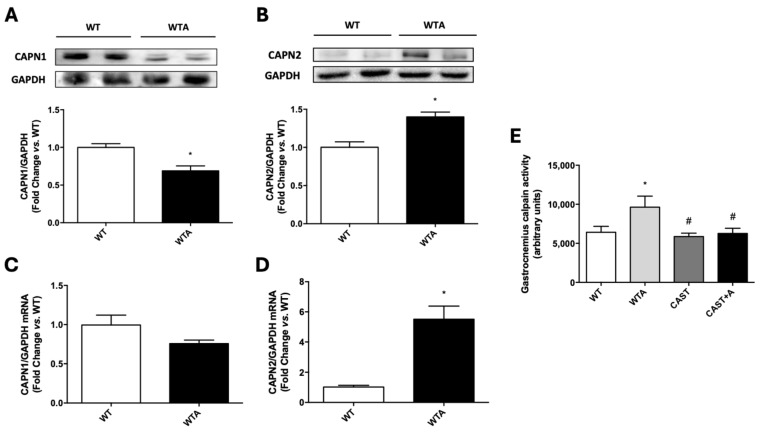
Calpain 1 and 2 content, expression and activity are modified in the gastrocnemius of adenine-fed mice. Wild-type (WT) were fed a standard or adenine-rich (WTA) diet for 4 weeks. (**A**,**B**): CAPN 1 (**A**) and 2 (**B**) protein content was evaluated by Western blot. An image of a representative blot is shown. The bars represent the normalized densitometric values of the blots against the endogenous control values versus WT. (**C**,**D**): Bar graphs show CAPN 1 (**C**) and 2 (**D**) mRNA expression, analyzed by RT-qPCR. Relative changes in mRNA content versus WT are plotted after normalization to total GAPDH content as an endogenous control. (**E**): In this panel, result from calpastatin-overexpressing mice, on standard (CAST) and adenine-rich diet (CAST+A) are also included. Gastrocnemius calpain activity (arbitrary units), evaluated by a fluorescence assay. All values are presented as the mean ± SEM. * *p* < 0.05 vs. WT; # *p* < 0.05 vs. WTA. n: 8–9 animals/group.

To start the analysis of the role of calpains in the genesis of these changes, calpain gastrocnemius levels were studied. Calpain 1 (CAPN 1) expression (protein and mRNA contents) decreased and calpain 2 (CAPN 2) expression increased in the gastrocnemius of mice during 4 weeks of adenine administration (Figure 5A–D). Moreover, there was a significant increase in calpain activity in the gastrocnemius of the 4-week adenine-fed mice (Figure 5E).

To demonstrate the role of calpains in the loss of muscle function in adenine-induced chronic renal damage, an experimental model of calpastatin overexpression was used. WT mice and their corresponding calpastatin-overexpressing littermates (CASTs) were subjected to the adenine-enriched diet for 4 weeks. CAST animals expressed the rabbit calpastatin transgene at the gastrocnemius level, and the presence of the transgene significantly decreased the adenine-induced augmented calpain activity at the gastrocnemius (Figure 5E).

Calpastatin overexpression improved the structural muscle changes observed in mice after 4 weeks of adenine administration, partially preventing the increase in muscle hind limbs mean diffusivity, T2 relaxation time and collagen deposition; as well as the decreased percentage of magnetization transfer (Figure 3A,B).

Furthermore, calpastatin overexpression partially prevented the muscle damage induced by adenine, as evidenced by reduced atrogin levels in the gastrocnemius (Figure 4A). Moreover, the increased mRNA expression of fibronectin, TNFα, and FABP4 observed after 4 weeks of adenine treatment was completely reversed in CAST+A mice (Figure 4B–D).

**Figure 6:**
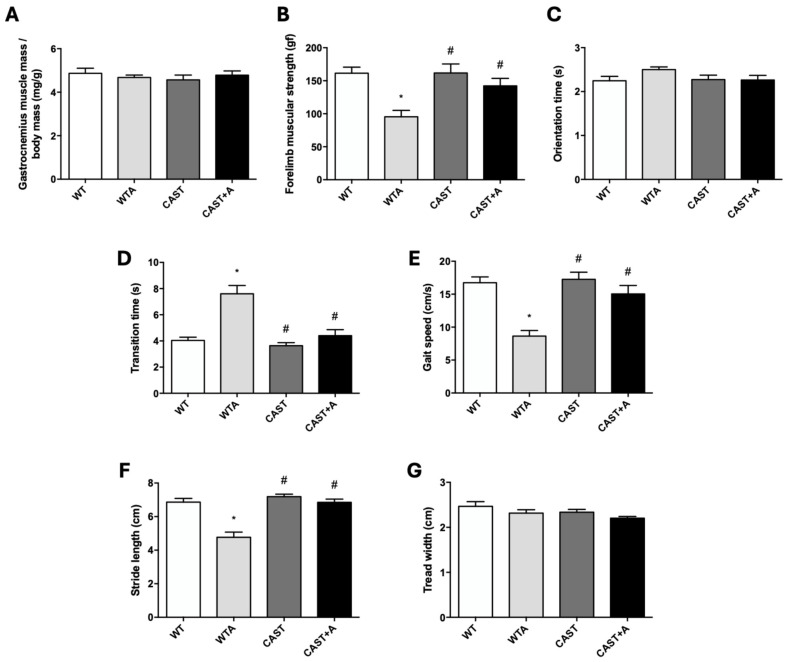
Calpastatin overexpression improves loss of muscle strength and physical performance of adenine-fed mice. Wild-type (WT) and calpastatin-overexpressing mice (CAST) were fed a standard or adenine-rich (WTA and CAST+A) diet for 4 weeks. (**A**): Muscle mass was estimated by analyzing the dry weight of gastrocnemius muscle and corrected by body weight (mg/g). (**B**): The bar graph shows the forelimb muscular strength (gf) measured by a grip strength test. (**C**–**E**): Bar graphs show orientation time (s) (**C**), transition time (s) (**D**) and gait speed (cm/s) (**E**), analyzed by a static bar test. (**F**,**G**): Bar graphs show stride length (cm) (**F**) and tread width (cm) (**G**) analyzed using a gait and stride analysis test. All values are presented as the mean ± SEM. * *p* < 0.05 vs. WT; # *p* < 0.05 vs. WTA. n: 12 animals/group.

**Figure 7:**
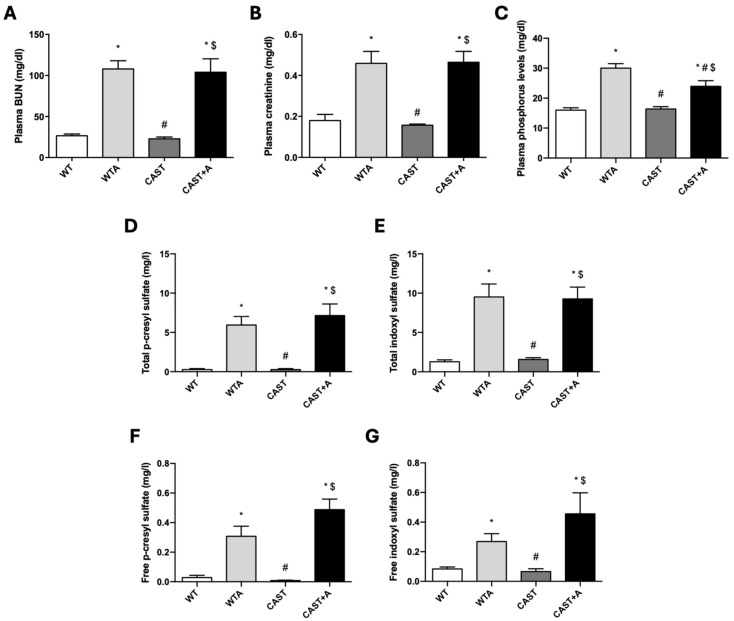
Calpastatin overexpression does not modify renal function in adenine-fed mice. Wild-type (WT) and calpastatin-overexpressing mice (CAST) were fed a standard or adenine-rich (WTA and CAST+A) diet for 4 weeks. (**A**–**C**): Bar graphs show the levels of BUN (blood urea nitrogen) (mg/dL) (**A**), plasma creatinine (mg/dl) (**B**), and plasma phosphorus (mg/dL) (**C**), analyzed by colorimetric assays. (**D**–**G**): Bar graphs show total p-cresyl sulfate (pCS) (**D**) and indoxyl sulfate (IS) (**E**); and free pCS (**F**) and IS (**G**) plasma levels (mg/L), analyzed by UHPLC-MS /MS. All values are presented as the mean ± SEM. * *p* < 0.05 vs. WT; # *p* < 0.05 vs. WTA; $ *p* < 0.05 vs. CAST. n: 7–8 animals/group.

Finally, calpastatin overexpression prevented loss of muscle strength and improved muscle function without altering renal function in adenine-fed mice. Gastrocnemius muscle mass was determined, but no significant changes were found in the four groups analyzed (Figure 6A). Regarding muscle strength (Figure 6B) and physical performance (Figure 6C–G), CAST+A animals partially reversed the loss of strength, significantly decreased transition time, and increased gait speed and stride length compared to WTA animals. On the other hand, no significant changes were observed in plasma urea (Figure 7A), creatinine (Figure 7B), or circulating levels of uremic toxins (Figure 7D–G), whereas phosphorus levels (Figure 7C) differed significantly between WTA and CAST+A animals.

In summary, adenine-induced CKD resulted in impaired muscle strength and physical performance, increased CAPN2 expression and CAPN activity, and enhanced markers of muscle damage, fibrosis, inflammation and adipogenesis. CAST overexpression partially or completely prevented these alterations, highlighting a functional link between CAPN activity and CKD-associated sarcopenia.

## 4. Discussion

The present results strongly support a key role for CAPN activity in CKD-related skeletal muscle impairment. Our study provides new evidence that CAPN2 upregulation is a key feature of CKD-related skeletal muscle impairment. While the involvement of CAPNs in muscle proteostasis has been previously described, their specific isoform contribution to CKD-related muscle deterioration remains poorly understood. We identified CAPN2 as the specific calpain isoform driving muscle loss in CKD. Furthermore, our results demonstrate that CAST overexpression enhances muscle strength and quality without modifying renal function parameters, indicating that the protective effects occur directly at the skeletal muscle level rather than as a secondary consequence of improved kidney function. These improvements are likely due to a reduction in CAPN-mediated damage mechanisms, including muscle atrophy, fibrosis, inflammation, and adipogenesis, highlighting the therapeutic potential of CAPN inhibition in preserving muscle quality in CKD.

Patients with CKD show high morbidity and mortality rates, largely due to cardiovascular and infectious complications. Uremia, resulting from impaired renal function, contributes to systemic damage, affecting multiple organs and reducing the quality of life and life expectancy [23]. In this context, frailty—originally described in geriatric populations—has emerged as a relevant clinical concept in CKD, with sarcopenia identified as one of its key components. Several studies have linked sarcopenia in CKD to uremic conditions and molecular mechanisms such as caspase activation, myostatin signaling, and the dysregulation of specific miRNAs [2]. CAPNs, known to contribute to muscle loss in other disease contexts [24], had not been extensively studied in CKD, particularly isoform-specific roles and systemic activity.

Previous findings from our research group showed that animals fed an adenine-rich diet for 2, 4, and 6 weeks develop impaired renal function [21]. Additionally, we observed that increased serum levels of phosphate, IS and pCS from week 2 onward are consistent with results from other studies using the same animal model [25,26]. In this context, animals with CKD induced by a 4- and 6-week adenine-rich diet showed reduced strength, impaired muscle function, and lower physical performance. Several studies examining skeletal muscle in adenine-induced CKD models have reported similar declines in muscle strength and function [27,28]. However, while other studies reported reductions in muscle mass in CKD models [29,30], we did not observe significant changes in gastrocnemius mass, possibly due to the shorter duration of our treatment. This aligns with reports indicating that muscle mass is not the sole determinant of strength and that factors such as fiber atrophy and impaired contractility contribute to functional decline [31,32]. Here, we analyzed CAPN content and markers of myogenic differentiation, muscle damage, fibrosis, inflammation, and adipogenesis after 4 weeks of adenine treatment—a time at which CKD was well established, and functional impairments were observed. Moreover, skeletal muscle comprises different fiber types, each with distinct susceptibility to atrophy and disease-related remodeling [33]. These differences likely explain the muscle-specific responses to CKD reported in the literature. In this study, we focused on the gastrocnemius muscle, which contains both oxidative and glycolytic fibers, allowing a comprehensive assessment of treatment effects.

CAPNs proteases participate in sarcomere turnover and cytoskeletal remodeling by cleaving proteins such as titin, dystrophin, nebulin, and desmin [15]. CAPN activation has been associated with disruption of myofibrillar structure and reduced muscle strength during atrophy [34]. Although there is controversy around CAPNs’ functions, imbalances in their expression or activity—whether up or downregulation—seem to trigger mechanisms leading to muscle damage. CAPN1 deficiency in knockout mice reduces proteolysis and favors hypertrophy of fast-twitch fibers [34], but CAPN1 may also have protective roles by limiting calcium-induced damage [35]. Its inhibition can impair muscle regeneration and promote atrophy [34], and deletion of capns1 leads to severe muscular dystrophy [35]. In CKD, muscle degradation is multifactorial and involves both reduced protein synthesis and enhanced proteolysis. In our model, the predominant mechanism appears to be increased CAPN activity, as CAPN2 upregulation coincided with functional impairment, fibrosis, inflammation, and adipogenic signaling. Notably, the concurrent decrease in CAPN1 and increase in CAPN2 expression suggests a compensatory shift among calpain isoforms, with CAPN2 potentially acting as the key driver of muscle damage in the uremic environment. Furthermore, increased cytoplasmic and mitochondrial levels of CAPN1 and CAPN2 have been linked to myotube atrophy [36], and CAPN activity is enhanced by calcium signaling channels [37]. Although intracellular Ca^2+^ concentration was not directly measured in this study, several CKD models have reported altered Ca^2+^ homeostasis and sarcoplasmic reticulum calcium release, conditions that may facilitate the activation of calcium-dependent CAPNs [26].

CAPN activity inhibition or silencing has been widely used to study their role in muscle damage [36,38]. In this study, partial inhibition of CAPN activity through overexpression of its endogenous inhibitor CAST prevented muscle strength loss and improved muscle function and physical performance in the adenine-induced CKD model. These results indicate that the loss of muscle strength observed in CKD cannot be explained solely by changes in muscle mass but is closely associated with calpain-mediated structural remodeling. Increased CAPN2 activity promotes atrophy, extracellular matrix accumulation, and pro-inflammatory and pro-adipogenic signaling, which together reduce muscle quality and contractile performance. Exposure to harmful stimuli in skeletal muscle triggers the activation of multiple molecular mechanisms involving key transcription factors such as FoxO1, FoxO3a, ATF4, C/EBPb, NF-kB, Stat3, and Smad2/3. These factors contribute to the upregulation and enhanced activity of proteolytic pathways, including CAPNs, thereby promoting muscle atrophy, inflammation, fibrosis and adipogenesis [3,39]. Accordingly, CAPN inhibition by CAST restored muscle function by reducing these pathological processes. These results suggest that CAPN2 overexpression may contribute to uremia-related muscle damage. To further investigate kidney damage and the role of CAPNs in muscle quality, NMRI scans were performed on a subset of animals [40]. Adenine-fed mice showed increased mean diffusivity (MD), reduced magnetization transfer (MT), and elevated T2 relaxation times—parameters associated with muscle atrophy, inflammation, edema, and connective tissue infiltration [41,42,43,44]. While CAST overexpression did not significantly reverse these changes, slight improvements were observed, though limited by the small sample size. These results underscore the importance of MRI as a complementary tool for assessing muscle quality beyond mass.

In the skeletal muscle of CKD animals, increased Sirius Red staining was observed in adenine-fed mice compared to controls, indicating higher collagen content and suggesting muscle tissue deterioration and reduced function due to uremia [7,45]. Additionally, fibronectin mRNA expression—a classical fibrosis marker—was elevated. However, mice overexpressing CAST showed significantly reduced fibrotic areas and lower fibronectin expression, suggesting that CAST overexpression may delay uremia-induced fibrosis.

Skeletal muscle atrophy is a common and serious complication of CKD. Various animal models, such as subtotal nephrectomy in rodents, have shown reduced muscle strength, decreased muscle mass, and decreased distance traveled, along with disrupted protein metabolism and increased expression of atrogenes [46]. One key enzyme involved in muscle atrophy is atrogin-1, which has been found to be upregulated in several CKD models [47,48]. Consistent with these findings, this study showed that feeding mice an adenine-rich diet for 4 weeks led to increased atrogin-1 levels in the gastrocnemius muscle.

In addition, skeletal muscle inflammation in the context of CKD has been studied in various animal models, showing increased expression of cytokines such as IL-1β, IL-4, IL-6, TNF-α, and IFN-γ, as well as macrophage markers like F4/80 [49,50]. These changes appear to be muscle-specific, occurring in muscles like the gastrocnemius but not in others like the soleus, suggesting fiber-type-dependent inflammation [51]. While systemic inflammation is a hallmark of CKD, skeletal muscle inflammation remains poorly explored. This study demonstrated increased TNF-α expression in the gastrocnemius of adenine-fed mice, consistent with previous findings [50,51]. Notably, CAST overexpression prevented the development of CKD-associated muscle inflammation, indicating that CAPNs may also contribute to local inflammatory processes in skeletal muscle under uremic conditions. These findings are consistent with previous studies demonstrating CAPN involvement in both local and systemic inflammatory responses across various pathophysiological contexts [52,53]. Specifically, CAPNs promote inflammation through activation of NF-κB signaling and stimulation of cytokine release [54], therefore CAST overexpression likely attenuates muscle inflammation and fibrosis by limiting calpain-dependent inflammatory signaling rather than acting as a direct anti-inflammatory factor.

To date, only a few studies have explored intramuscular fat accumulation in the context of CKD. In dialysis patients, increased intramuscular fat and reduced muscle area have been linked to systemic inflammation and poor physical function [55]. Notably, in sarcopenia, muscle strength and function can decline without a loss in muscle mass, and increased fat infiltration and reduced muscle quality are key diagnostic features. CKD studies have shown that lower muscle quality is associated with reduced strength and performance [56], supporting the findings of this study. In CKD animal models, increased intramuscular fat and activation of fibro-adipogenic progenitor (FAP) cells have also been observed [7]. Consistent with this, our model showed elevated FABP4 mRNA expression in the gastrocnemius. CAPNs have been proposed to modulate muscle adipogenesis as their dysregulation may influence preadipocyte differentiation and promote lipid accumulation [39,57]. This could occur via disruption of signaling pathways such as Wnt/β-catenin, which is crucial for cell fate regulation [58]. In this study, CAST overexpression prevented adipogenesis in the gastrocnemius of adenine-fed mice, suggesting that CAPNs play a role in CKD-associated intramuscular fat accumulation. Further studies in animal and cellular models are needed to clarify the underlying mechanisms and support the development of effective therapies.

Importantly, CAST overexpression did not lead to improvements in renal function parameters in adenine-fed mice, including serum creatinine or uremic toxin levels, suggesting that the observed benefits in muscle quality and function are not secondary to improved kidney function but rather reflect an intrinsic effect of CAPN inhibition in skeletal muscle. These findings support the hypothesis that CAPNs directly contribute to muscle deterioration in CKD and that their modulation may provide therapeutic benefits even in the presence of ongoing renal dysfunction.

One of the limitations of our study is that both CAST overexpression and capns1 deletion occur globally, not just in the kidney, so that other organs may be affected by the inhibition of CAPN1 and 2. As a proposal for future research, it would be interesting to use a model in which CAST overexpression or the specific deletion of capns1 occurs exclusively at the kidney level.

Altogether, our results demonstrate that CAPNs contribute to multiple aspects of muscle deterioration in CKD, including atrophy, fibrosis, inflammation, and fat infiltration. Inhibition of CAPN activity via CAST improves muscle performance and reduces pathological changes, supporting its potential as a therapeutic strategy for preserving muscle quality in CKD. Future studies should not only measure muscle mass but also comprehensively assess muscle quality, incorporating markers of fibrosis, inflammation, fat accumulation, and functional capacity in both preclinical and clinical contexts.

## 5. Conclusions

This study demonstrates that calpains, particularly CAPN2, play a central role in muscle deterioration associated with chronic kidney disease (CKD). Partial inhibition of calpain activity through calpastatin (CAST) overexpression effectively prevented muscle strength loss, improved physical performance, and reduced muscle fibrosis, inflammation, and adipogenesis, without altering renal function. These findings identify CAPN2 as a key contributor to CKD-related sarcopenia and support its potential as a therapeutic target. Further studies are needed to validate these results in clinical settings and to explore the role of calpain inhibition in preventing frailty and preserving muscle quality in CKD patients.

## Data Availability

The original data presented in the study are openly available in Zenodo at 10.5281/zenodo.17307417.

## Abbreviations

The following abbreviations are used in this manuscript:

CAPN	Calpain
CAST	Calpastatin
CKD	Chronic kidney disease
IS	Indoxyl sulfate
MD	Mean diffusivity
MT	Magnetization transfer
NMRI	Nuclear magnetic resonance imaging
pCS	p-cresyl sulfate
WT	Wild-type
